# The distinctive role of menthol in pain and analgesia: Mechanisms, practices, and advances

**DOI:** 10.3389/fnmol.2022.1006908

**Published:** 2022-10-05

**Authors:** Ziping Li, Haoyue Zhang, Yigang Wang, Yize Li, Qing Li, Linlin Zhang

**Affiliations:** ^1^The Graduate School, Tianjin Medical University, Tianjin, China; ^2^Department of Anesthesiology, Tianjin Medical University General Hospital, Tianjin, China

**Keywords:** menthol, pain, transient receptor potential channel, opioid receptor, analgesia

## Abstract

Menthol is an important flavoring additive that triggers a cooling sensation. Under physiological condition, low to moderate concentrations of menthol activate transient receptor potential cation channel subfamily M member 8 (TRPM8) in the primary nociceptors, such as dorsal root ganglion (DRG) and trigeminal ganglion, generating a cooling sensation, whereas menthol at higher concentration could induce cold allodynia, and cold hyperalgesia mediated by TRPM8 sensitization. In addition, the paradoxical irritating properties of high concentrations of menthol is associated with its activation of transient receptor potential cation channel subfamily A member 1 (TRPA1). Under pathological situation, menthol activates TRPM8 to attenuate mechanical allodynia and thermal hyperalgesia following nerve injury or chemical stimuli. Recent reports have recapitulated the requirement of central group II/III metabotropic glutamate receptors (mGluR) with endogenous κ-opioid signaling pathways for menthol analgesia. Additionally, blockage of sodium channels and calcium influx is a determinant step after menthol exposure, suggesting the possibility of menthol for pain management. In this review, we will also discuss and summarize the advances in menthol-related drugs for pathological pain treatment in clinical trials, especially in neuropathic pain, musculoskeletal pain, cancer pain and postoperative pain, with the aim to find the promising therapeutic candidates for the resolution of pain to better manage patients with pain in clinics.

## Introduction

Chronic pain affecting more than 30% of people worldwide (Cohen et al., 2021) with enormous personal and financial burdens. Concerns about the adverse effects, interactions, and potential for abuse of analgesic drugs have forced the search for alternative methods to manage pain effectively and safely. Although several therapies have been proposed, inadequate knowledge of the underlying pathophysiological mechanisms is one of the reasons for the current lack of pain relief.

Menthol have been used for pain relief since ancient times, first described by Hippocrates (Sprengell, 1708) and Galen (Siegel, 1970). Hippocrates considered mint as a cooling agent for peripheral pain. Galen expanded the use of menthol even further, so that existing topical analgesic containing menthol were named “Menthol Galen-Pharma.” Menthol is a cyclic monoterpene alcohol, which is derived primarily from aromatic plants. It is a natural compound with three asymmetric carbon atoms and, thus occurs as four pairs of optical isomers namely (+)- and (-)-isomenthol, (+)- and (-)- menthol, (+)- and (-)-neumenthol, (+)- and (-)-neoisomenthol. The principal form of menthol found in nature is (-)-menthol (L-menthol), and the main supply is obtained naturally. The Nobel Prize for chemistry was awarded to Ryoji Noyori in 2001 for the work on asymmetric catalysis for menthol chemical synthesis (Kamatou et al., 2013).

Menthol is one of the most important flavorings additives besides vanilla and citrus (Kamatou et al., 2013) and is used as a cooling and/or flavors enhancing ingredient in medicines, cosmetics and insecticides, confectionery, chewing gum, liqueurs, toothpaste, shampoos, and soaps (Patel et al., 2007; Kolassa, 2013). Menthol exhibits unique, multiple, and paradoxical sensory effects when applied externally to the skin or mucous membranes. menthol application at low doses produces a cooling sensation, whereas at higher doses, it evokes burning, irritation, and pain (Wei and Seid, 1983; Wasner et al., 2004; Namer et al., 2005; Proudfoot et al., 2006). In practice, various topical over-the-counter products containing menthol for pain relief have concentrations ranging 5−16% (320−1024 mM) (Oz et al., 2017).

Several *in vitro* and *in vivo* studies have demonstrated menthol’s biological properties, such as its analgesic, antibacterial, antifungal, anesthetic, and osmotic enhancement, as well as chemoprophylaxis and immunomodulatory effects (Kamatou et al., 2013). The multiple physiological effects of menthol appear to depend on its interaction with a variety of receptors throughout the body. It has been proposed to sensitize transient receptor potential cation channel subfamily M member 8 (TPRM8) and transient receptor potential cation channel subfamily V member 3 (TRPV3), desensitize transient receptor potential cation channel subfamily A member 1 (TRPA1) and transient receptor potential cation channel subfamily V member 1 (TRPV1), stimulate κ-opioid systems, and enhance central glutamate dependent gate control mechanisms (Green and McAuliffe, 2000; Macpherson et al., 2006; Proudfoot et al., 2006; Zanotto et al., 2008). This complex and unique effect of menthol has contributed to its potential as a promising area of pain research. This review summarizes the current pre-clinical results and clinical practice evidence on menthol in analgesic research.

## Analgesic mechanism of menthol

### Menthol and transient receptor potential family

Transient receptor potential (TRP) channels are a family of proteins which are present in nociceptors and allow organisms to transduce environmental signals that produce pain (Rosenbaum et al., 2022), of which, the discovery is a Nobel physiology and medicine-awarded subject in 2021. Currently, menthol is considered to be the activity regulator of TRPM8, TRPA1 and TRPV1, etc., in the TRP family, and thus menthol and its chemically modified derivatives are currently highlighted again.

#### Transient receptor potential cation channel subfamily M member 8

In general, menthol selectively acts on TRPM8, a non-selective calcium-permeable channel expressed in a subset of sensory neurons in the dorsal root ganglion (DRG) and trigeminal ganglion (Galeotti et al., 2002; McKemy et al., 2002), which are thought to be the main molecular sensor of cold somatosensation in human body (activation temperature is 8−28°C) (Andersen et al., 2014; Olsen et al., 2014). In addition to menthol and low temperature, TRPM8 channels can be activated by a variety of stimuli (Izquierdo et al., 2021), including the menthol analog icilin (Colburn et al., 2007; Izquierdo et al., 2021), changes in voltage (Fernández et al., 2011; Raddatz et al., 2014) and extracellular osmotic pressure (Parra et al., 2014; Quallo et al., 2015).

Transient receptor potential cation channel subfamily M member 8 participates in cold signal transmission in two general ways: one that transduces innocuous cool temperatures to trigger cool sensation, behavioral thermoregulation, and analgesia; and another that transmits nociceptive signals to trigger more overtly nocifensive behaviors (Chung and Caterina, 2007). For the body in physiological state, TRPM8 predominantly activate the first, but in pathological conditions such as nerve injury or inflammation, TRPM8 mainly plays the second role (Marwaha et al., 2016; De Caro et al., 2019). To understand how interactions among different concentrations of menthol, different chemical or physical stimuli, and the structure of the TRPM8 channel determines pain states, it is necessary to understand that there are three important properties of their function that may be affected to modulate their gating (closing and opening of the ion conduction pathway): *activation*, *sensitization* and *desensitization* (Rosenbaum et al., 2022). To be more specific, in physiological states, low to moderate concentrations of menthol activate TRPM8 receptors to produce a feeling of coolness; at higher concentration, menthol could induce cold allodynia, and cold hyperalgesia mediated by TRPM8 sensitization; following nerve injury or chemical stimuli, menthol activates TRPM8 to mediate relief of mechanical allodynia and thermal hyperalgesia; long-term local exposure to menthol also desensitizes TRPM8-sensitive fibers. Based on the results of experiments with topical menthol solutions applied to rodents, the dividing line between “low” and “high” concentrations we refer to here is 10% wt/vol or 640 mM.

##### TRPM8 activation

Almost all the analgesic potency of menthol comes from its activation of TRPM8. It is worth noting that the term “analgesia” specifically refers to the relief of noxious heat, chemical stimuli, and mechanical allodynia. In patch-clamp recordings, menthol activated wild-type TRPM8 with the half maximal effective concentration (EC_50_) of 185.4 ± 69.4 μM (Xu et al., 2020). TRPM8 activation evokes an influx of Ca^2+^ into the primary sensory neuron, leading to its activation and the propagation of action potentials. The activation of TRPM8 by menthol has been extensively studied in a variety of *in vivo* rodent models. Proudfoot et al. (2006) demonstrated that peripherally (4 mM) or centrally (200 nM) applied menthol, in a model of neuropathic pain (chronic compressive nerve injury, CCI) marked reverse behavioral reflex sensitization to noxious heat and mechanical stimulation. In addition, sensitization specific thermal and mechanical analgesia induced by TRPM8 activation has also been observed in focal demyelination of the sciatic nerve (Proudfoot et al., 2006), Complete Freund’s adjuvant (CFA) intra plantar injection (Proudfoot et al., 2006; Pan et al., 2012), and chemical stimulus (such as capsaicin, acrolein or cinnamaldehyde) (Proudfoot et al., 2006; Liu et al., 2013). Systemic or topical application of menthol can dose-dependently increase pain threshold in rodents in hot plate tests (Galeotti et al., 2002; Klein et al., 2010; Liu et al., 2013). In addition, direct evidence that TRPM8 may explain all the analgesic activity of menthol was provided by Liu et al. (2013) using genetic and pharmacological approaches in mice: the gene deletion and selective inhibitor (AMG2850) of TRPM8 completely eliminated the TRPM8-dependent analgesia on chemical stimuli, noxious heat, and inflammation. It is worth noting that the activation of TRPM8 can be regulated by neurotrophic factors (Lippoldt et al., 2013, 2016), phosphatidylinositol bisphosphate (PIP_2_) (Premkumar et al., 2005; Rohács et al., 2005), Ca^2+^-independent phospholipase A2 (iPLA2) and polyunsaturated fatty acids (Andersson et al., 2007), forming a complex network.

It is important to note that in the above study, menthol produced TRPM8-dependent analgesia of acute and inflammatory pain over a wide range of concentrations and administration routes (oral, intraperitoneal, intraplantar or topical), from estimated systemic levels of 60−120 μM, to a topical concentration in the molar range (30% wt/vol) (Liu et al., 2013). This means, that this concentration range may be lower than the reported TRPM8 activation concentration; means that cause analgesia in the sensitized pain state with lower doses of TRPM8 activation.

##### TRPM8 sensitization

In the physiological state, high concentrations of menthol can induce TRPM8 sensitization and promote the development of cold allodynia and cold hyperalgesia by excessively lowering the cold pain threshold. Menthol-induced cold hypersensitivity is believed to primarily rely on direct sensitization of TRPM8 on Aδ and C-fibers (Andersen et al., 2014). Numerous studies indicate that high concentration of menthol can cause hyperalgesia in cold perception in rodents (>10% wt/vol or >640 mM for mice topically, Tajino et al., 2007), which may make menthol valuable in pre-clinical screening of analgesic agents for cold hyperalgesia (Rossi et al., 2006; Tajino et al., 2007), and it provides a theoretical basis for the establishment of experimental cold hyperalgesia pain model in human with topically high concentration of menthol (>30% wt/vol for healthy subjects, Hatem et al., 2006).

In contrast to the peripheral sensitization of TRPM8 neurons by menthol under physiological conditions, the actual conditions leading to hypersensitivity under pathological conditions are complex (Andersen et al., 2014). In the periphery, nociceptive cold fibers can become sensitized upon axonal degeneration leading to dysregulation of TRP channel expression, resulting in a more excitable phenotype (Gold et al., 2003). Preclinical studies have shown that TRPM8 expression in sensory neurons (DRG and superficial dorsal horn) is increased with the development of hypersensitivity to mechanical, thermal, and cold stimuli in rats with CCI (Proudfoot et al., 2006; Colburn et al., 2007; Frederick et al., 2007; Xing et al., 2007; Su et al., 2011, 2017) and L5 and L6 spinal nerve ligation (SNL) (Katsura et al., 2006; Patel et al., 2014; Koh et al., 2016). Overexpression of TRPM8 has been linked to development of cold allodynia. Evaporative cooling of acetone may represent an inherently less toxic cold stimulus, so it is common to assess cold sensitivity by measuring the response of laboratory animals to acetone application. Colburn et al. (2007) used CCI rodent model to observe significant sensitivity to acetone application. In contrast, TRPM8 gene deletion mice showed no significant increase in acetone sensitivity at any time after ligation (Rimola et al., 2021). This sensory disturbance was also successfully reversed by TRPM8 mRNA antisense (Su et al., 2011). Administration of oxaliplatin and other chemotherapeutic agents can lead to chemotherapy-induced peripheral neuropathy (CIPN), which is characterized by cold abnormal pain and mechanical hyperalgesia. Oxaliplatin treatment has been shown to induce an increase in TRPM8 expression in DRG (Kawashiri et al., 2012; Mizuno et al., 2014; Yamamoto et al., 2018), and that oxaliplatin-induced cold hypersensitivity is diminished in TRPM8 knockout mice (Descoeur et al., 2011). Recently updated studies have shown that the activity of the TRPM8 but not the activity of any other member of the TRP channel family is transiently increased after oxaliplatin treatment (Rimola et al., 2021). These changes will increase the body’s sensitivity to cold and thus reduce the perception of pathological pain, which may represent a protective effect of self-pain reduction in biological evolution.

##### TRPM8 desensitization

Desensitization occurs when the channel becomes refractory to an activating stimulus by adopting a conformational state where the passage of ions is not allowed. In the context of the nociceptive system, desensitization may function as a protective mechanism in which hyperexcitation or cell death by ion-dependent signaling pathways is avoided (Rosenbaum et al., 2022). To be specific, the activity of the cold- and menthol-activated TRPM8 diminishes over time in the presence of extracellular Ca^2+^ (Yudin et al., 2011; Diver et al., 2019), which is manifested by a decrease in the cooling effect over time when exposed to menthol, along with a decrease in TRPM8 activity (Cliff and Green, 1994; Reid and Flonta, 2001; Matsu-ura et al., 2006; Daniels et al., 2009). In addition, menthol can cause a short-lasting cross-desensitization of other irritant chemicals-induced stimulus including capsaicin (Cliff and Green, 1996; Green and McAuliffe, 2000; Naganawa et al., 2015), citric acid (Morice et al., 1994; Plevkova et al., 2013), cinnamaldehyde (Zanotto et al., 2008; Klein et al., 2011), and nicotine (Dessirier et al., 2001; Fan et al., 2016; Huynh et al., 2020, etc.), that is, a reduction in irritation evoked by compounds other than itself (Dessirier et al., 2001).

#### Transient receptor potential cation channel subfamily A member 1

However, abnormal pain induced by high menthol concentration still exists experimental fact that is difficult to be explained by sensitization of TRPM8. Fan et al. (2016) demonstrated that mice showed a concentration-dependent oral aversion of menthol (>100 μg/mL or >640 mM), and that gene deletion of TRPM8 did not reduce this aversion. Lemon et al. (2019) conducted a brief-access exposure tests in mice to measure reduction in orosensory avoidance behaviors to aqueous menthol solutions and showed that oral aversion to menthol was reduced in mice deficient for TRPA1 but not TRPM8.

Due to the low selectivity of menthol (Macpherson et al., 2006; Vogt-Eisele et al., 2007), it can also activate TRPA1 (Karashima et al., 2007; Xiao et al., 2008), which is a member of the TRP family with TRPM8. Expression of TRPA1 is limited in a subset of nociceptive neurons of the trigeminal ganglion and DRG (Story et al., 2003; Kobayashi et al., 2005) and is a sensor for a wide variety of environmental stimuli, such as intense cold activation temperature is <18°C (Story et al., 2003), close to 8°C, the reported threshold of noxious cold, Wahren et al. (1989), Tillman et al. (1995), and Harrison and Davis (1999) irritating compounds, reactive chemicals, and endogenous signals associated with cell injury (Talavera et al., 2020) to elicit protective responses. Although both respond to cold stimuli, TRPM8 and TRPA1 are activated by harmless cooling and harmful cold (Caterina et al., 1997; Bandell et al., 2004), respectively. Irritating compounds that directly activate TRPA1 and may cause pain in humans include cinnamaldehyde, allyl isothiocyanate, allicin, formalin (Bandell et al., 2004; Bautista et al., 2005; Moran et al., 2011), and bradykinin as inflammatory mediators (Bandell et al., 2004; Obata et al., 2005). TRPA1 has been implicated in several painful and inflammatory conditions and is considered a promising potential target for the development of, amongst others, analgesic, antipruritic and anti-inflammatory pharmacotherapeutics (Andrade et al., 2012; Koivisto et al., 2012; Preti et al., 2015). The irritating properties of menthol have been shown to be associated with this activation of TRPA1 (Karashima et al., 2007; Fan et al., 2016). In concert with the sensitivity of TRPM8 for menthol, Karashima et al. (2007) showed bell-shaped dose-response curve of menthol to channels by whole-cell and single-channel recordings of heterologously expressed TRPA1, demonstrate a bimodal sensitivity of TRPA1 to menthol, further proved that menthol acts as an effective agonist of TRPA1 at relative low concentrations (100−300 μM), but acts as an antagonist at higher concentrations [≥300 mM (Karashima et al., 2007)] by causing reversible channel blocking, although this high-concentration blocking effect was only found in rodent models, not human models (Macpherson et al., 2006; Karashima et al., 2007; Xiao et al., 2008; Lemon et al., 2019).

#### Transient receptor potential cation channel subfamily V member 1

Menthol also causes analgesic effects through TRPM8-independent mechanisms, such as the TRPV1, which is involved in the transmission and modulation of nociception, as well as the molecular integration of diverse painful stimuli (Huang et al., 2002; Cui et al., 2006). The activators of TRPV1 are noxious heat [activation temperature is >42°C, in the range of perception that shifts from innocuous warmth to noxious heat; the reported threshold of noxious heat is 55°C (Patapoutian et al., 2003)], acidic pH, capsaicin (the irritating compound in hot chili peppers), and allyl isothiocyanate (Everaerts et al., 2011). TRPV1 is a non-selective cation channel; when it is activated, Na^+^ and Ca^2+^ flowing into the cell to depolarize nociceptive neurons, leading to action potential firing and finally leads to a painful, burning sensation (Caterina et al., 1997). In particular, the functions of TRPV1 and TRPA1 interlink with each other to a considerable extent. This is especially clear in relation to pain and neurogenic inflammation where TRPV1 is co-expressed on the vast majority of TRPA1-expressing sensory nerves and both integrate a variety of noxious stimuli (Fernandes et al., 2012). At the same time, TRPV1 also has a bimodal characteristic similar to TRPA1, which is activated by low menthol concentration and inhibited by high menthol concentration. Nguyen et al. (2021) observed that TRPV1 was activated by 3 mM menthol (HEK293T cells expressing rat TRPV1) and Takaishi et al. (2016) found *in vitro* that the TRPV1 current was completely inhibited at higher concentrations (>10 mM, HEK293T cells expressing human TRPV1), and this concentration is sufficient to activate TRPM8 and induce anti-nociceptive effects in response to thermal and mechanical stimuli in practice. This may be related to an important feature of TRPV1, which is rapid desensitization after activation, making the channel refractory to further stimulation (Szallasi and Blumberg, 1999). Thus, TRPV1 agonist that can cause desensitization and TRPV1 antagonists can both be considered as promising novel types of analgesics in therapeutic regimen (Gavva et al., 2008; Gunthorpe and Chizh, 2009). This suggests that the desensitization/inhibition of menthol on TRPV1 may be one of the potential mechanisms to explain the analgesic effect of menthol, and menthol and its derivatives could be promising molecules to develop TRPV1 antagonists (Takaishi et al., 2016).

In addition, menthol activates TRPV3, which specifically expressed in keratinocytes (skin cells) to produce a “warm” sensation at innocuous temperatures (activation temperature is about 34−38°C) (Peier et al., 2002; Xu et al., 2002; Macpherson et al., 2006), which would seem to explain one of the curious effects of menthol administration is that it can make human subjects report spontaneous sensations of warmth (Green, 1985; Hatem et al., 2006). *In vitro* heterologous TRPV3 expressing CHO cells, 0.5−2 mM menthol induced TRPV3 currents in a concentration dependent manner, while TRPA1 and TRPV1 currents were inhibited. Also, the sensitization to TRPV3 currents was observed with repeated application of 0.5 mM menthol (Macpherson et al., 2006). Although TRPV3 and TRPV1 share over 40% identity (Peier et al., 2002; Smith et al., 2002; Xu et al., 2002), little research has been done on the structural features that make them functionally so different.

As mentioned above, menthol induces analgesia through several different TRP mechanisms. For example, the simultaneous administration of menthol in response to capsaicin-induced hyperalgesia may also be involved: activation and desensitization of TRPM8 by menthol, activation of TRPA1 by menthol at higher concentrations, activation of TRPV1 and TRPA1 by capsaicin, inhibition of TRPV1 by menthol, and other mechanisms. The resulting analgesic effect of menthol should be the net effect of this complex network of mechanisms. Rodent models of menthol and its analogs are shown in Table 1.

**TABLE 1:** Rodent models of menthol and its analogs.

Rodent models	Compound	Route of administration	Concentration	Effects	References
CCI, SD rats	L-Menthol	Subcutaneously	10%	Cold hypersensitivity exacerbated	Zuo et al., 2013
CCI, Wistar rats	Icilin	Topical	<500 μM	Behavioral-reflex sensitization reversed in thermal and mechanical tests	Proudfoot et al., 2006
			>5000 μM	Hyperalgesia effects	
		Intrathecal injection	<10 nM	Behavioral-reflex sensitization reversed in thermal and mechanical tests	
	L-Menthol	Topical	4 mM		
		Intrathecal injection	200 nM		
SNL, SD rats	Icilin	Topical	1 mM	Nociceptive behaviors enhanced	Ji et al., 2007
SNL, SD rats	Icilin	Topical	10−200 μM	Not affected tactile allodynia or thermal hyperalgesia, but cold allodynia increased	Hagenacker et al., 2014
		Intrathecal injection	0.1 nM−1 μM		
SNL, SD rats	L-Menthol	Topical	10% and 40%	Alleviate cooling hypersensitivity; no significant effect in mechanical withdrawal thresholds	Patel et al., 2014
	M8-Ag	Subcutaneously	30 mg⋅kg^–1^	Reverse behavioral hypersensitivity to cooling	
Hot-plate and abdominal constriction, Swiss albino mice	L-Menthol	Oral	3−10 mg⋅kg^–1^	The pain threshold increased dose-dependently	Galeotti et al., 2002
		Intracerebroventricularly	10 μg		
Thermal Paw Withdrawal, SD rats	L-Menthol	Topical	0.1−10%, and 40%	Withdrawal latency increased	Klein et al., 2010
Von Frey Paw Withdrawal, SD rats				Only the 40% menthol group was significantly different from all other groups indicating allodynia	
Two-temperature preference test, SD rats				Low cold sensitivity at high concentration (10% and 40%); cold allergy at low concentrations (0.01−1%)	
Cold plate test, SD rats				Cold antinociceptive effect of 40% menthol	
CFA-induced inflammatory pain, CD-1 mice	L-Menthol	Intraperitoneal injection	100 mg⋅kg^–1^	Thermal and mechanical hypersensitivity reduced	Pan et al., 2012
Formalin-Induced Spontaneous Nociceptive Behavior, CD-1 mice				Nociception decreased	
Hot plate test, C57BL/6 mice	L-Menthol	Oral	10 mg⋅kg^–1^	Approximately doubled paw withdrawal latencies at both temperature (52°C or 55°C)	Liu et al., 2013
Hot plate test, C57BL/7 mice		Topical	30%	Analgesic effect	
Hot plate test, C57BL/8 mice		Intraperitoneal injection	20 mg⋅kg^–1^	Tail flick latencies increased	
Capsaicin-induced nocifensive behavior, C57BL/8 mice		Injected into the plantar surface of the hind paw	20 μM	Mechanical hyperalgesia inhibited	
Capsaicin-induced mechanical hyperalgesia, C57BL/8 mice			1 mM	Nocifensive behavior inhibited	
Acrolein-induced TRPA1-dependent nocifensive behavior, C57BL/8 mice					
SNL, SD rats	L-Menthol	Injected into the plantar surface of the hind paw	1%, 10%, and 40%	Cold hypersensitivity alleviated, no change in receptive field size was observed or in heat, dynamic brush, or electrically evoked responses	Patel et al., 2014
TRPM8^+/+^ mice	Icilin	Intraperitoneal injection	60 mg⋅kg^–1^	Robust jumping response	Colburn et al., 2007
CCI, C57/B6 mice	L-Menthol	Topical	40 μL	Licking duration increased, also displayed behaviors such as flinching of the paw and brushing of the affected area	Caspani et al., 2009
WT, C57BL/6 mice	L-Menthol	Oral	>50 μg/mL	Consumed less mentholated water and more plain water	Fan et al., 2016
WT, C57BL/6 mice	L-Menthol	Oral	>0.7 mM	Oral aversion	Lemon et al., 2019
PpIX phototoxicity pain, NIH SWISS mice	L-Menthol	Topical	2% and 16%	Pain behavior reduced	Wright et al., 2018
CFA, Wistar rats	L-Menthol	Intraperitoneal injection	100 mg⋅kg^–1^	Anti-hyperalgesia properties and no negative locomotor side effects	Hilfiger et al., 2021
AmmVIII intraperitoneally, C57BL/6 mice	L-Menthol	Subcutaneously	1 mM	Analgesic effects of mechanical hypersensitivity	Gaudioso et al., 2012

CCI, chronic constriction injury; CFA, complete Freund’s adjuvant; SD rats, Sprague-Dawley rats; SNL, spinal nerve ligation; TRPA1, transient receptor potential ankyrin 1.

### Menthol and glutamate receptor

Activation of TRPM8 by menthol analog icilin is reported to produce analgesia by activating central inhibitory pathways (Chung and Caterina, 2007; Dhaka et al., 2007), which make use of inhibitory group II/III metabotropic glutamate receptors in the spinal cord (mGluRs). Numerous studies revealed that the mGluRs play a major role in modulatory central nervous system pathways and have been suggested to have pharmacological antinociceptive implications in inflammatory, neuropathic, and acute pain (Fisher et al., 2002; Simmons et al., 2002; Chen and Pan, 2005).

Proudfoot et al. (2006) demonstrated that intrathecal administration of group II and group III mGluRs antagonists prevented the reversal of thermal and mechanical sensitization of intrathecal menthol (200 nM) and topical icilin (200 μM) after CCI in rats, from which they hypothesized that: the activation of TRPM8 by menthol or icilin in a subpopulation of afferents results in the release of glutamate from central synapses, which then acts through inhibitory group II/III mGluRs located either pre-synaptically on injury-activated nociceptive afferents or perhaps also post-synaptically on dorsal-horn neurons, thereby attenuating neuropathic sensitization. These findings highlight the possible value of menthol and its analogs as TRPM8 activators and downstream central targets of TRPM8 action as novel analgesics for development.

### Menthol and opioid

Transient receptor potential cation channel subfamily M member 8-mediated menthol antinociception was found to be dependent on activation of the endogenous opioid pathway (Galeotti et al., 2002; Liu et al., 2013; Patel et al., 2014), and intensive opioid receptors stimulation can also suppresses TRPM8 activity in DRG neurons, lead to the internalization of TRPM8 to pain relief (Shapovalov et al., 2013), suggesting that the two share a common pathway for modulating pain sensation in general. The non-selective opioid receptor antagonist naloxone (Liu et al., 2013; Shapovalov et al., 2013), and the selective κ-antagonist nor-NBI significantly reduced the TRPM8-dependent analgesia of acute and inflammatory pain (Galeotti et al., 2002), whereas the selective μ-antagonist CTOP and δ1-antagonist 7-benzylidenenal-trexone (BNTX) and δ2-antagonist naltriben did not prevent menthol-induced antinociception, therefore, the menthol-induced TRPM8-dependent analgesia is more likely mediated by the activation of the central κ-opioid system, excluding the involvement of μ and δ opioid systems (Galeotti et al., 2002).

Opioid tolerance and opioid-induced hyperalgesia is common in patients who have for chronically treated with opioids (Colvin et al., 2019). Cold allodynia is also a common complaint of patients chronically treated with opioids. In contrast with the findings that acute morphine triggers TRPM8 internalization (Shapovalov et al., 2013) and Gong and Jasmin (2017) demonstrated in rodent models that chronic morphine administration induced cold hyperalgesia and up-regulation of TRPM8. However, knockdown, or selective blocking of TRPM8 can reduce cold hyperalgesia caused by morphine. Iftinca et al. (2020) further confirmed that chronic morphine enhances cold hypersensitivity by increasing excitability and reduction desensitization in TRPM8-expressing neurons through activation of μ-opioid-receptor-protein kinase C beta (MOR-PKCβ) signaling pathway, which can be reversed by a follow-up treatment with the inverse non-selective opioid receptor antagonist naloxone (Shapovalov et al., 2013).

In addition to TRPM8, a strong connection has been suggested between the TRPV1 and the opioid pathway. Chronic administration of morphine up-regulates TRPV1 expression in spinal cord, DRG and sciatic nerve (Niiyama et al., 2007; Chen et al., 2008; Vardanyan et al., 2009); blocking TRPV1 by SB366971 (Chen et al., 2008) and capsazepine (Nguyen et al., 2010) or destructing TRPV1-expressing sensory neurons by resiniferatoxin (Chen and Pan, 2006) significantly inhibited morphine tolerance in rodents. TRPV1 has also been reported to be a physiological regulator of opioid receptors through a β-arrestin2 and PKA-dependent manner (Bao et al., 2015; Scherer et al., 2017; Basso et al., 2019). Thus, it is conceivable that menthol-mediated modulation of TRPV1, and thus regulation of opioid pathways affording a promising avenue to improve the therapeutic profile of opioid medications.

In addition, studies have shown that TRPA1, which is involved in the formation of harmful signals, significantly attenuates its association with μ-opioid receptors in the DRG, spinal cord and brain areas under the mediation by calcium-activated calmodulin in formalin-induced inflammatory pain and CCI-induced neuropathic pain in mice (Cortés-Montero et al., 2020).

**FIGURE 1:**
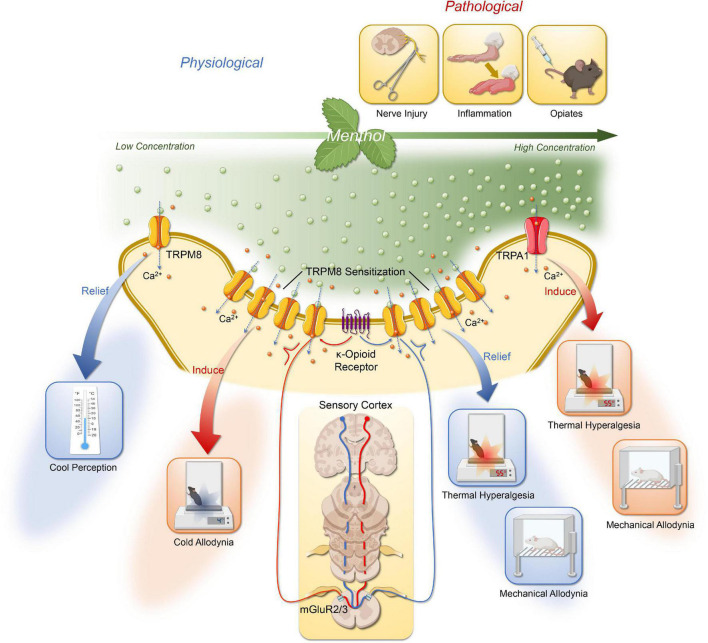
The possible mechanisms of menthol action on the DRG or trigeminal ganglion *via* TRPM8 and TRPA1. The effects of menthol are concentration dependent. Low to moderate concentrations of menthol activate TRPM8 in the DRG and trigeminal ganglion to produce a cooling sensation; at higher concentration, menthol could induce cold allodynia, and cold hyperalgesia mediated by TRPM8 sensitization; following nerve injury or chemical stimuli, menthol activates TRPM8 to mediate relief of mechanical allodynia and thermal hyperalgesia. Its analgesic effect is also dependent on the activation of central group II/III mGluR with endogenous κ-opioid signaling pathways. In addition, the thermal and mechanical allodynia of high concentrations of menthol is associated with its activation of TRPA1. Created with BioRender.com.

In conclusion, existing studies demonstrate cross-talk interactions between opioid pathways and TRP, these mechanisms could provide a basis for better understanding general analgesia associated with opiate administration and hyperalgesia associated with abstinence. We illustrate the possible mechanisms of menthol action on the DRG or trigeminal ganglion (Figure 1).

### Other potential mechanisms

In addition to the above mentioned, a number of different kinds of ligand and voltage-gated ion channels for menthol mediated analgesia have been proposed, and these mechanisms may partially explain the TRP-independent mechanisms are responsible for menthol analgesia.

#### Voltage-gated Na^+^ channels

Menthol has local anesthetic activity. Galeotti et al. (2001) found that menthol significantly reduced electrically induced contractions of rat phrenic hemidiaphragm in a dose-dependent manner; the number of stimuli necessary to provoke the rabbit conjunctival reflex increased in a dose-dependent manner. By recording heterologously expressed whole-cell sodium inward currents in rat neuronal and human skeletal muscle, Haeseler et al. (2002) used patch-clamp to demonstrate that menthol acts like the local anesthetic lidocaine to suppress whole cell Na^+^ currents with an IC_50_ of 571 and 376 μM for neuronal cells and skeletal muscle fibers, respectively. In another electrophysiological study, Gaudioso et al. (2012) conclude that menthol inhibits Nav1.8, Nav1.9, and TTX-sensitive Na^+^ channels in a concentration-, voltage-, and frequency-dependent manner. It is worth mentioning that eucalyptol (an agonist of TRPM8, 1 mM), and BCTC (a TRPM8 antagonist, IC_50_ = 0.8 μM) had no effect on the above Na^+^ currents, indicating that the blocking effect of menthol on Na^+^ channels is independent of TRPM8. Another study on cortical neurons reported that menthol (250 μM) dampens the generation of action potentials in TRPM8 knock-out mice and in the presence of a TRPM8 blocker, suggest that menthol can modulate voltage-gated sodium channels in cortical neurons through a TRP-independent pathway (Pezzoli et al., 2014).

#### Voltage-gated Ca^2+^ channels

Several reports have demonstrated that menthol modulates the functional properties of voltage-gated Ca^2+^ channels. *In vitro* studies have shown that menthol blocks Ca^2+^ influx in Helix neurons (Swandulla et al., 1986), cultured DRG cells (Swandulla et al., 1987), LA-N-5 human neuroblastoma cells (Sidell et al., 1990), leech neurons (Dierkes et al., 1997), guinea-pig ileal smooth muscle, rat and guinea-pig atrial and papillary muscle, rat brain synaptosomes and chick retinal neurons (Hawthorn et al., 1988). In addition, menthol decreased the muscle contractures of tracheal (Ito et al., 2008; Wang et al., 2016), bronchial (Wright et al., 1997), vas deference (Filippov et al., 2009; Vladymyrova et al., 2011), colon (Amato et al., 2014), and aortae, mesenteric, coronary arteries (Cheang et al., 2013) smooth muscle by inhibiting of L-type voltage-gated calcium channels. Ramos-Filho et al. (2014) proved that menthol (300 μM) or nifedipine (1 μM) inhibited carbachol and electrical field stimulation induced contractions in both wild type and TRPM8 knockout bladder strips, conclude that menthol inhibits smooth muscle contraction by blocking L-type Ca^2+^ channels in a manner independent of TRPM8 activation. Menthol not only limits Ca^2+^ influx through voltage-gated Ca^2+^ channels, but also induces Ca^2+^ release from intracellular storage pools (endoplasmic reticulum and Golgi) in a TRP-independent manner (Palade, 1987; Tsuzuki et al., 2004; Lu et al., 2006; Mahieu et al., 2007; Wondergem and Bartley, 2009; Neumann and Copello, 2011; Melanaphy et al., 2016). Based on these findings, Melanaphy et al. (2016) revealed that the vasorelaxation effect of menthol and other TRPM8 agonists on isolated rat tail artery myocytes is the net effect of non-specific inhibition of L-type Ca^2+^ channels and activation of Ca^2+^ release in the sarcoplasmic reticulum. These findings will promote the understanding of the vascular effects of TRPM8, particularly the well-known hypotensive effects of menthol (Sun et al., 2014), and may also have translational implications (Melanaphy et al., 2016).

In addition, menthol can potently inhibit K_*v*_7.2/3 channels (a voltage-gated K^+^ channel) isoforms (IC_50_ = 289 μM) and their mediated M currents. These mechanisms may be helpful in explaining TRPM8-mediated cooling and analgesic effects (Vetter et al., 2013).

#### GABA_*A*_ receptors

In the central nervous system, γ-amino butyric acid (GABA) is the major inhibitory neurotransmitter. In responding to an action potential, it is released from the pre-synaptic button into the synaptic cleft and acts on either ionotropic GABA_*A*_ receptors or metabotropic G-protein coupled GABA_*B*_ receptors. Among them, the activation of GABA_*A*_ receptors causes hyperpolarization and eventually the suppression of neuronal activity (Fritschy et al., 2012; Sigel and Steinmann, 2012). In practice, GABA_*A*_ are the main targets of many sedatives and general anesthetics (Millan, 2003; Hemmings et al., 2005). Menthol acts as a potent positive allosteric modulator at GABA_*A*_ (Hall et al., 2004; Corvalán et al., 2009). Menthol can enhance the GABA_*A*_-mediated currents in the spinal dorsal horn (Pan et al., 2012), CA1 pyramidal neurons of the hippocampus (Zhang X. B. et al., 2008), periaqueductal grey (PAG) neurons in midbrain slices (Lau et al., 2014) and *Xenopus oocytes* (Watt et al., 2008) *in vitro*. In particular, the TRPM8 agonist icilin, the non-selective TRPM8/TRPV1 antagonist Capsazepine or the TRPA1 antagonist HC-030031 did not affect menthol potentiation of GABA_*A*_ receptor-mediated currents in PAG neurons in midbrain slices from rats, indicating that the menthol actions occur independently of TRP channel activation (Lau et al., 2014). Just like propofol, which acts on a similar site in the GABA_*A*_ receptor and has a general anesthetic effect, menthol has been shown to be a moderate anesthetic with an EC_50_ of 23 μM, about 10-fold less potent anesthesia than propofol (Thorup et al., 1983; Krasowski et al., 2001). While menthol was far less potent than propofol in producing anesthesia its toxicity is minimal (Thorup et al., 1983) and therefore point to menthol as a lead compound for the development of novel GABAergic modulatory drugs (Watt et al., 2008).

#### Nicotinic acetylcholine receptors

Menthol is also often added to tobacco products because of the “coolness” of smoke, which is believed to bring pleasure to smokers (Werley et al., 2007); at the same time, menthol may reduce respiratory irritation response found in tobacco smoke (Hans et al., 2012). These applications have been proved to be related to possible functional interaction between menthol and nicotinic acetylcholine receptors (nAChRs) (Dessirier et al., 2001; Ruskin et al., 2008; Willis et al., 2011; Hans et al., 2012; Ashoor et al., 2013a). The nAChRs are cation-permeable ion channel-receptor complex activated by the neurotransmitter acetylcholine, which play important roles in several physiological functions and pathological conditions (Dani and Bertrand, 2007; Dineley et al., 2015). *In vitro* electrophysiologic studies showed that menthol was a negative allosteric regulator of α4β2 subtypes of nAChRs (Hans et al., 2012); menthol can even desensitize α3β4 subtype of nAChRs through allosteric mechanism (Ton et al., 2015); and menthol can also inhibit the α7 subtype of nAChRs non-competitively (Ashoor et al., 2013a). In addition, Henderson et al. (2016) demonstrated that chronic menthol induces cell-type-selective up-regulation of α4* subtype of nAChRs and also alters midbrain dopamine neuron function; more importantly, since menthol plus nicotine can produce greater reward-related behavior than nicotine alone (Henderson et al., 2017), this may explain the difficulty in quitting smoking (Foulds et al., 2010), whereas menthol can enhance smoking behavior and stimulate adverse effect of smoking on health as an additive to tobacco products (Kabbani, 2013; Wickham, 2015).

Menthol is also an allosteric non-competitive inhibitor of the 5-hydroxytryptamine type 3 (5-HT_3_) receptor (Heimes et al., 2011; Ashoor et al., 2013b; Walstab et al., 2014; Ziemba et al., 2015), which may explain its effective antiemetic properties.

It is worth noting that Liu et al. (2013) pointed out in their study on the role of TRPM8 in menthol induced analgesia that, in addition to activating TRPM8 channels, the concentration in the experiment (60−120 μM for systemic use and 30% wt/vol for external use) was sufficient or far more than the concentration required for pharmacological effects on other potential targets involved in menthol induced analgesia mediator mentioned above. However, any residual analgesic effect of menthol was not observed in TRPM8 knockout mice, which may imply these alternative targets: 1. may have little or no interaction with menthol to affect pain perception; 2. may only play a role in menthol analgesia under specific situations; 3. may have specific analgesic effect for other menthol isoforms (Liu et al., 2013). Therefore, further studies are needed to explore the interaction between menthol and these other potential alternative targets in analgesic effects.

## Menthol in clinical practice

Over the past 15 years, a large number of menthol and its derivatives have been described by large pharmaceutical companies, small biotech companies and the academic community, but only a few have made it into clinical trials and even fewer have completed clinical trials.

### Menthol-induced human cold hyperalgesia pain model

Under pathophysiological conditions, normally innocuous skin cooling can induce pain. This cold hyperalgesia (allodynia) is a striking symptom in patients with neuropathic pain (Lindblom and Verrillo, 1979; Wahren and Torebjörk, 1992; Woolf and Mannion, 1999; Baron, 2000). Translational surrogate models of pain, allodynia and hyperalgesia make it possible to evaluate the efficacy of drugs in pre-clinical settings and contribute to improved screening, diagnosis and treatment strategies for patients by applying these models in healthy volunteers to narrow the gap between animals and patients (Arendt-Nielsen and Yarnitsky, 2009). However, pathological animal models such as CCI, SNL and CIPN that induce neuropathic pain or hyperalgesia are often irreversible and invasive and cannot be used as human replacement models *per se*. As above, there have been numerous pre-clinical studies using menthol to establish cold hypersensitivity. In view of this, topical high concentration of L-menthol is the only human experimental pain model established to investigate the mechanism of cold hyperalgesia (Andersen et al., 2015). The work of Andersen et al. (2014) summarized the topical application of menthol in humans as a transformational model for cold abnormal pain and hyperalgesia. According to their findings, most of the menthol was applied to the volar forearm (Wasner et al., 2004; Namer et al., 2005; Hatem et al., 2006; Flühr et al., 2009); the highest menthol concentration that can actually be triggered effectively is about 40% (Wasner et al., 2004, 2008; Namer et al., 2005, 2008; Seifert and Maihöfner, 2007; Altis et al., 2009; Flühr et al., 2009; Binder et al., 2011; Mahn et al., 2014), but a 30% menthol ethanol solution proved to be the ideal formulation because of its effectiveness and safety (Hatem et al., 2006); the most common spontaneous sensation reported by subjects during application was coldness or mild pain; all studies reviewed consistently found a significant increase in cold pain threshold in healthy volunteers (Andersen et al., 2015). Human studies on topical menthol induced cold pain are summarized in Table 2.

**TABLE 2:** Human models of menthol and its products.

Compound	Route of administration	Indication	Effects	Clinical trial number	References
10 mM menthol	Oral	Healthy subjects	Transiently desensitize capsaicin-sensitive fibers	NA	Proudfoot et al., 2006
40% Menthol	Topical	Healthy subjects	Topical menthol induced cold allodynia	NA	Wasner et al., 2004, 2008; Namer et al., 2005, 2008; Seifert and Maihöfner, 2007; Altis et al., 2009; Flühr et al., 2009; Binder et al., 2011; Mahn et al., 2014
30% Menthol	Topical	Healthy subjects	30% Topical menthol can induce cold hyperalgesia with effectively and safely	NA	Hatem et al., 2006
10% Menthol	Topical	Healthy subjects	No significant to histamine-induced itch and pain sensation	NA	Yosipovitch et al., 1996
Tetracaine gel with vs. without menthol	Topical	Healthy subjects	Improved the analgesic efficacy of the tetracaine gel in part through enhanced percutaneous permeation	NA	Liu et al., 2005
Menthol smokers vs. non-menthol smokers	NA	Healthy subjects	Less pain and pain-related functional interference	NA	Kosiba et al., 2019
0%, 10%, 20% and 30% menthol	Topical	Healthy subjects	The concentration-dependent grading response elicits a range of sensations	NA	Wright et al., 2019
Topical pain-relieving patch (containing 6% menthol) vs. placebo	Topical	Mild to moderate pain condition	Significant relief of pain	NA	Gudin et al., 2020
40% Menthol	Topical	Upper limb amputees	Induce hypersensitivity	NA	Vase et al., 2013
40% Menthol vs. placebo	Topical	Healthy subjects, Trans-cinnamaldehyde-induced hyperalgesia	*No Results Posted*	NCT02653703	Andersen et al., 2016
5% Menthol cream vs. placebo	Topical	After photodynamic therapy	*No Results Posted*	NCT02984072	
Menthol (Biofreeze) vs. placebo	Topical	Acute LBP	Significant relief of pain	NA	Zhang J. et al., 2008
Menthol (Biofreeze) vs. placebo	Topical	Non-radicular mechanical neck pain	*No Results Posted*	NCT03012503	
3.5% Menthol gel vs. inert placebo gel	Topical	OA	No significant improvement	NA	Topp et al., 2013
Menthol (Biofreeze) vs. placebo	Topical	OA	*No Results Posted*	NCT03888807, NCT04351594, NCT01565070	
Menthol (Biofreeze) vs. placebo	Topical	EIMD	Menthol may reduce the sensation of muscle soreness and may influence recovery of lower-body power	NA	Gillis et al., 2020
Menthol (Biofreeze) vs. placebo	Topical	DOMS	Significant relief of pain associated with increased corticospinal inhibition	NA	Stefanelli et al., 2019
3.5% menthol gel vs. application of ice	Topical	DOMS	Decreased perceived discomfort to a greater extent and permitted greater tetanic forces to be produced.	NA	Johar et al., 2012
3.5% menthol gel vs. inert placebo gel	Topical	Soft tissue injuries	Significant relief of pain	NA	Airaksinen et al., 2003
3% menthol + ibuprofen vs. gel ibuprofen gel alone/diclofenac gel	Topical	Soft tissue injuries	Significant relief of pain	NA	Wade et al., 2019
Mentholated cream only vs. MC containing oxygenated glycerol triesters	Topical	Acute musculoskeletal pain	The combination resulted in a gradual reduction in pain, lower VAS scores, and greater improvements in lifestyle and mobility improvements	NCT01387750	Taylor et al., 2012
3.5% Menthol patch vs. placebo patch	Topical	Mild to moderate musculoskeletal strain	Significant relief of pain	NA	Higashi et al., 2010
1% Diclofenac/3% menthol gel vs. inert placebo gel	Topical	Acute ankle sprain	No significant improvement	NCT02100670	Lai et al., 2017
Menthol (Biofreeze) vs. placebo	Topical	CTS	*No Results Posted*	NCT01716767	
Menthol (Biofreeze) vs. placebo	Topical	CTS	Significant relief of pain	NA	Sundstrup et al., 2014
Neat peppermint oil (containing 10% menthol)*	Topical	Postherpetic neuralgia	Significant relief of pain	NA	Davies et al., 2002
Menthol (Biofreeze) vs. placebo	Topical	MPS	Increase in the mouth opening size	NA	Yaman et al., 2021
1% Menthol cream	Topical	CIPN, PMPS	Significant relief of pain	NA	Fallon et al., 2015
1% Menthol cream*	Topical	CIPN	Significant relief of pain	NA	Cortellini et al., 2017
5% Menthol cream*	Topical	CIPN	Significant relief of pain	NA	Brid et al., 2015
7.5% Methyl salicylate/2% menthol lotion vs. placebo	Topical	CIPN	*No Results Posted*	NCT01855607	
3.5% Menthol gel vs. inert placebo gel	Topical	CIPN	*No Results Posted*	NCT04276727	
Mannitol + menthol cream vs. menthol cream	Topical	PDPN	*No Results Posted*	NCT02728687	
No cooling vs. menthol-containing cooling bandage vs. ice containing cold pack	Topical	Undergoing anterior cruciate ligament reconstruction	Positive subjective comfort feedback	NA	Engelhard et al., 2019
Menthol 10% solution vs. menthol 0.5% solution	Topical	Migraine	Significant relief of pain	NA	Borhani Haghighi et al., 2010
6% Menthol gel (STOPAIN)	Topical	Migraine	Significant relief of pain	NCT01687101	St Cyr et al., 2015; Kayama et al., 2018
40% Menthol	Topical	Histamine-induced itch	An inhibitory effect on histaminergic itch	NA	Andersen et al., 2017; Engelhard et al., 2019
Menthol based aromatic therapy vs. placebo	NA	Second trimester genetic amniocentesis	No significant improvement	NA	Hanprasertpong et al., 2015

* Case reports. CTS, carpal tunnel syndrome; CIPN, chemotherapy-induced peripheral neuropathy; DOMS, delayed-onset muscle soreness; EIMD, exercise-induced muscle damage; LBP, low back pain; MPS, Masticatory myofascial pain syndrome; OA, osteoarthritis; PDPN, painful diabetic peripheral neuropathy; PMPS, post-mastectomy pain syndrome; VAS, Visual Analog Scale.

### Musculoskeletal pain

Knowledge about the feasibility of topical menthol for musculoskeletal pain dates back centuries, as topical menthol has been widely used to treat muscle soreness (Silva, 2020). The most common musculoskeletal pain is low back pain, which affects 30% to 40% of the general population and is the leading cause of disability (Hartvigsen et al., 2018). Using a randomized controlled design, Zhang J. et al. (2008) determined to add Biofreeze (Performance Health Inc., Export, PA, United States), a locally applied pain-killing agent containing menthol, to the chiropractic treatment plan for patients with low back pain, with significant pain relief effect. However, there were no significant changes in Roland-Morris Disability Questionnaire (RMDQ), heart rate variability, or LBP electromyography (EMG) compared with the control group, and further studies with a larger sample size are needed to confirm the findings (Zhang J. et al., 2008). In addition, NCT03012503 has compared the effects of Biofreeze with placebo on neck pain after cervical massage with chiropractic, but no results have been published to date.

Osteoarthritis (OA) is the main cause of disability in the elderly (Glyn-Jones et al., 2015). Topp et al. compare the ability to complete functional tasks and knee pain while completing functional tasks among 20 patients with knee OA after topical application of either 3.5% menthol gel or an inert placebo gel, but no differences were found in functional tasks or pain (Topp et al., 2013). NCT03888807 attempted to determine the effects of Biofreeze vs. placebo on walking gait characteristics and walking pain in patients with bilateral knee OA but announced early termination due to difficulty in enrolling subjects. In addition, NCT03888807, NCT04351594, and NCT01565070 have been evaluating the effect of Biofreeze on knee OA, but no results have been obtained.

Post-exercise myalgia and delayed onset muscle soreness (DOMS) are common symptoms after exercise and physical activity. Stefanelli et al. (2019) found that DOMS reduced corticospinal excitability and after the administration of menthol-based topical analgesic, there was a reduction in pain, which was accompanied by increased corticospinal inhibition. Although ice application is considered the traditional method for post-exercise pain relief, Johar et al. (2012) demonstrated that compared to ice, the topical menthol-based analgesic decreased perceived discomfort to a greater extent and permitted greater tetanic forces to be produced, and the effectiveness of ice for acute injury has not been proven in any clinical trials and may even be harmful (Bleakley et al., 2004). Airaksinen et al. (2003) showed that topical menthol gels not only provide excellent pain relief, but also promote recovery in patients with exercise-related soft tissue injuries. Higashi et al. (2010) reported in a double-blind randomized controlled trial that a single 8-h patch containing methyl salicylate and menthol significantly alleviated pain associated with mild to moderate muscle strain in these adult patients compared to patients receiving a placebo patch. NCT02100670 is a multicenter, randomized, double-blind, placebo-controlled, parallel-group trial performed to evaluate topical 1% diclofenac/3% menthol gel in treating ankle sprain, but no significant improvement was observed with topical 1% diclofenac/3% menthol gel compared with placebo, 1% diclofenac, or 3% menthol gel in treating pain from ankle sprain (Lai et al., 2017).

Carpal tunnel syndrome is a neuromuscular disease caused by increased pressure on the median nerve of the wrist, with common symptoms include wrist and hand pain, paresthesia, thenar muscle weakness and loss of flexibility (Padua et al., 2016). A three-blind randomized placebo-controlled crossover trial demonstrated that topical menthol significantly reduced pain intensity compared with placebo in abattoir workers with chronic pain and carpal tunnel syndrome symptoms (Sundstrup et al., 2014).

### Neuropathic pain

Neuropathic pain is defined as pain caused by a lesion or disease of the somatosensory nervous system and is a major therapeutic challenge. Common causes of neuropathic pain include cerebrovascular accident, multiple sclerosis or spinal cord injury, or peripheral nervous system, for example, painful diabetic neuropathy, postherpetic neuralgia, or surgery (Boyd et al., 2019). The first reported use of menthol in neuropathic pain dates to 1870, when menthol oil was successfully used to treat neuropathic facial pain.

Chemotherapy-induced peripheral neuropathy is a severe and painful adverse reaction of cancer treatment in patients. CIPN that occurs during chemotherapy, sometimes requiring dose reduction or cessation, impacting on survival (Colvin, 2019). Menthol activated TRPM8 channels are a promising therapeutic target in CIPN (Colvin, 2019). In Fallon et al. (2015), in a proof-of-concept study, determined that 82% of evaluable patients had an improvement in their total pain scores after 4−6 weeks of treatment with topical 1% menthol cream, and 50% had a clinically relevant reduction in pain scores of at least 30%. Cortellini et al. (2017) reported the remarkable treatment with menthol cream of a male patient with a history of metastatic colon cancer and previous chemotherapies who had neuropathy that impaired his quality of life and limited further chemotherapy. Another clinical study (NCT01855607, as shown in Table 2) assessed whether 6 weeks of treatment with topical menthol twice daily would reduce CIPN in patients have completed adjuvant or neo-adjuvant Taxane based breast cancer therapy or Oxaliplatin based colon cancer chemotherapy. Recently, a phase II study (NCT04276727, as shown in Table 2) used a special brain scan called functional magnetic resonance imaging (fMRI) to help determine whether topical menthol therapy has potential for CIPN patients. In a word, CIPN patients may benefit from the use of menthol, either during the treatment of patients complain of subjective improvement, lead to a better quality of life, and can be implemented without interruption of chemotherapy and effective chemotherapy dose delivery, which in turn lead to longer survival, is likely to be an effective potential palliative treatment option.

Neuropathy is one of the most common long-term complications of diabetes, characterized by altered thermal, mechanical, and chemical sensation. Of three large, clinic-based studies from Europe, the prevalence of diabetic neuropathy varied from 23–29% (Young et al., 1993; Tesfaye et al., 1996; Cabezas-Cerrato, 1998). Preclinical studies have shown that there was an increase in TRPV1-mediated currents but a decrease in TRPM8-mediated currents in DRG isolated from diabetic hyperalgesia mice. Therefore, menthol, which has the effect of targeting TRPV1 or TRPM8, may be a useful approach to treat pain associated with diabetic neuropathy (Pabbidi and Premkumar, 2017). There has been a randomized, double-blind, placebo-controlled crossover trial of the efficacy and safety of menthol topically alone or in combination with mannitol in the relief of diabetic neuropathy (NCT02728687), with no results have been reported.

In addition, Davies et al. (2002) reported successful relief of postherpetic neuralgia using topical peppermint oil in combination with other interventions.

### Others

Management of postoperative pain relieve suffering and leads to earlier mobilization, shortened hospital stay, reduced hospital costs, and increased patient satisfaction (Gupta et al., 2010). Engelhard et al. (2019) observed a beneficial effect of cooling by a menthol-containing bandage during the rehabilitation phase after anterior cruciate ligament reconstruction. The reduction of muscle cross section within 30 days after surgery was prevented by menthol dressings, which highly contributed to rehabilitation success after 90 days of therapy and can reduce painkiller consumption (Engelhard et al., 2019).

Genome-wide association analysis have revealed that TRPM8 is associated with susceptibility to migraine without aura (Freilinger et al., 2012), and reduced TRPM8 expression (rs10166942 carriers) helps reduce migraine risk in humans (Gavva et al., 2019), which may provide the rationale for the topical menthol as an alternative treatment option for migraine patients. A randomized, triple-blind, placebo-controlled, crossed-over study demonstrated the efficacy, safety, and relative tolerability of cutaneous application of 10% menthol solution in the treatment of migraine without aura (Borhani Haghighi et al., 2010). An open-label pilot study showed that 52% of subjects experienced at least one statistically significant improvement in migraine severity 2 h after using topical menthol (St Cyr et al., 2015). Due to the limitations of these studies, larger placebo-controlled clinical trials are needed.

Pruritus and pain are closely related but distinct sensations. There is a broad overlap between pain- and itch-related peripheral mediators and/or receptors, and there are astonishingly similar mechanisms of neuronal sensitization in the peripheral and central (Ikoma et al., 2006). Analgesia and antipruritic therapy should have common targets. In 1995, Bromm et al. found that topical menthol had a central inhibitory effect on histamine-induced pruritus of the lower left arm in 15 healthy male volunteers (Bromm et al., 1995). Andersen et al. (2017) conducted a similar study and got the same conclusion, further verified the antipruritic effect of menthol like doxepin. Using a combination of pharmacologic, genetic, and mouse behavioral assays, Palkar et al. (2018) found that the inhibition of pruritus by menthol requires TRPM8 channels or intact and functional TRPM8-expressing afferent neurons. Menthol and its analogs that rely on TRPM8 channels are a promising target for the development of pruritus therapies, and further clinical trials could significantly improve the management of pruritus.

## Conclusion

We summarize the progress of menthol in pain and analgesia research based on existing evidence and demonstrate the important role of menthol in clinical analgesia. Although TRPM8 may explain the analgesic effect of menthol, the molecular mechanism between upstream and downstream has not been fully elucidated, and the exact association between menthol, TRP family, mGluRs and endogenous κ-opioid signaling pathway has not been established. Furthermore, menthol interactions with TRPA1 and other targets may have pro-nociceptive and inflammatory effects. In fact, topical menthol treatment is often accompanied by skin irritation, and inhalation of menthol can aggravate asthma in some patients, which may be the role of TRPA1 (Bautista et al., 2006; Zhang X. B. et al., 2008; Caceres et al., 2009). This is very similar to the action of beta-adrenergic receptors in the circulatory system. Therefore, it is urgent to develop TRPM8-specific drugs to replace menthol in analgesic and anti-irritant therapy, to prevent these adverse effects and allow more effective analgesic treatment.

## Author contributions

LZ, ZL, and HZ designed and collected the literatures, and wrote the manuscript. YW and YL collected some literatures and created the figure and tables. LZ and QL revised the manuscript. All authors contributed to the article and approved the submitted version.
